# A systematic literature review of reported challenges in health care delivery to migrants and refugees in high-income countries - the 3C model

**DOI:** 10.1186/s12889-019-7049-x

**Published:** 2019-06-14

**Authors:** Julia Brandenberger, Thorkild Tylleskär, Katrin Sontag, Bernadette Peterhans, Nicole Ritz

**Affiliations:** 1 University Children’s Hospital Basel, Migrant Health Service, University of Basel, Spitalstr.33, Basel, Postbox CH 4031 Switzerland; 2 Swiss Tropical and Public Health Institute, P.O. Box, CH-4002, Basel, Switzerland; 3 University of Basel, P.O. Box, CH-4003,, Basel, Switzerland; 4 Paediatric Emergency Department, Inselspital, University of Bern, Bern, Switzerland; 5 Centre for International Health, University of Bergen, Bergen, Norway; 6 Department of Social Sciences, Subject Area Cultural Anthropology, University of Basel, Basel, Switzerland; 7 University Children’s Hospital Basel, Pediatric Infectious Disease and Vaccinology, University of Basel, Basel, Switzerland; 8 Royal Children’s Hospital Melbourne, Department of Pediatrics, University of Melbourne, Parkville, Australia

**Keywords:** Asylum, Confidence, Continuity of care, Communication, Interpreter, Immigrant, Quality of care, Translator, Refugee, Trust

## Abstract

**Background:**

Migrants and refugees have important health needs and face inequalities in their health status. Health care delivery to this patient group has become a challenging public health focus in high income countries. This paper summarizes current knowledge on health care delivery to migrants and refugees in high-income countries from multiple perspectives.

**Methods:**

We performed a systematic literature review including primary source qualitative and quantitative studies between 2000 and 2017. Articles were excluded if the study setting was in low- or middle-income countries or focused on skilled migration. Quality assessment was done for qualitative and quantitative studies separately. Predefined variables were extracted in a standardized form. Authors were approached to provide missing information.

**Results:**

Of 185 identified articles, 35 were included in the final analysis. We identified three main topics of challenges in health care delivery: communication, continuity of care and confidence. All but one study included at least one of the three main topics and in 21/35 (60%) all three topics were mentioned. We further developed the 3C model and elaborated the interrelatedness of the three topics. Additional topics identified showed that the specific regional context with legal, financial, geographical and cultural aspects is important and further influences the 3C model.

**Conclusions:**

The 3C model gives a simple and comprehensive, patient-centered summary of key challenges in health care delivery for refugees and migrants. This concept is relevant to support clinicians in their day to day practice and in guiding stakeholders in priority setting for refugee and migrant health policies.

**Electronic supplementary material:**

The online version of this article (10.1186/s12889-019-7049-x) contains supplementary material, which is available to authorized users.

## Background

The number of migrants worldwide has risen by over 105 million, or by 69% since 1990 [1]. In 2017, an estimated 68.5 million individuals were displaced globally, including 25.4 million Refugees [2]. The health needs of this increasing population of migrants and refugees are a global challenge for health care systems. Access to high quality health care is particularly important for these individuals as they face unequal medical treatment opportunities. Rising numbers of migrants and refugees in host countries put migrant’s and refugee’s health on the public health agenda [3].

The vision of the United Nations 2030 Sustainable Development Goals is to leave no one behind and to strive for peace and reduction of inequity [4]. For migrants and refugees, ways to improve health care delivery are detailed by the World Health Organization (WHO) which include the need for patient-centered and intercultural approaches [5].

Even though the largest group of displaced individuals and refugees are hosted in resource poor countries, the health needs of migrants and refugees need to be addressed in high income countries. As one strategy to improve migrant health care delivery, the European Union started an initiative called the “migrant-friendly hospital project” in 2002. The approach focused on improving interpreting services, providing migrant-friendly information and training staff in cultural competence [6]. Based on the experience of this project the “Amsterdam Declaration” identified the need for a comprehensive training of health care providers to understand the specific requirements of migrants and refugees [7].

It is key to adequately train health care providers in health care delivery to migrants and refugees. In this paper, we review the literature on knowledge, perceptions and attitudes of migrants, refugees and healthcare providers regarding health care delivery in high-income countries. We aim to develop a comprehensive, patient-centered model summarizing the main challenges described.

## Methods

A mixed method literature review was performed [8] guided by the preferred reporting items for systematic reviews and meta-analyses (PRISMA [9]) and the mixed methods research synthesis framework (MMRS [10]). The systematic literature review was done using three separate search strings for primary source qualitative and quantitative studies published between 1 Jan 2000 and 31 Dec 2017.

First, the search included medical subject headings (MeSH) terms (“delivery of health care” AND “health services”) AND (“migrant*” OR MeSH term “refugees”) in all databases of Web of Science (Clarivate Analytics) including Web of Science Core Collection, Current Contents Connect, Data Citation Index, MEDLINE, Russian Science Citation Index, SciELO Citation index. Second, the search term “health care delivery” AND “migrant*” and third “health care delivery” AND “refugee*” were used in the Web of Science Core Collection to allow for a less restricted search. The two terms “migrant*” and “refugee*” were used to allow for search results that were as broad as possible including asylum seekers, resettlement refugees, recognized refugees, undocumented refugees and migrants. There was no language restriction. Identified studies were excluded according to the following criteria: a) study setting in low- or middle-income countries, b) skilled migration, c) no focus on health care delivery to migrants and/or refugees, d) migrants for medical reasons, e) insufficient description of design and methods. Inclusion and exclusion of studies were discussed between JB and NR.

Quality assessment was done by JB and NR and disagreements discussed until consensus was reached. For cross-sectional studies a modified Newcastle-Ottawa scale, as recommended by the Cochrane Collaboration [11] was used and for qualitative studies the critical appraisal check list for qualitative research from the Oxford-based Critical Appraisal Skills Programme [12]. For the completeness of the review we also included good practice reports and expert opinion articles.

As heterogeneity of the included studies was substantial, a qualitative synthesis approach was chosen. A thematic analysis was done for both qualitative and quantitative data. (Additional file 1: Figure S1) [10]. Variables were extracted in a standardized form, including the following: title, first author, publication date, setting, timeframe of the study, described challenges (literal approach). If information was unavailable, authors were contacted to provide missing information. In a stepwise approach, the described challenges were condensed into topics using a narrative approach. Views of health workers and patients were compared where necessary. The interrelation of the main challenges was visualized in the final model.

The corresponding author had full access to all the data in the study and had final responsibility for the decision to submit for publication. There was no funding source for this study.

## Results

**Fig. 1:**
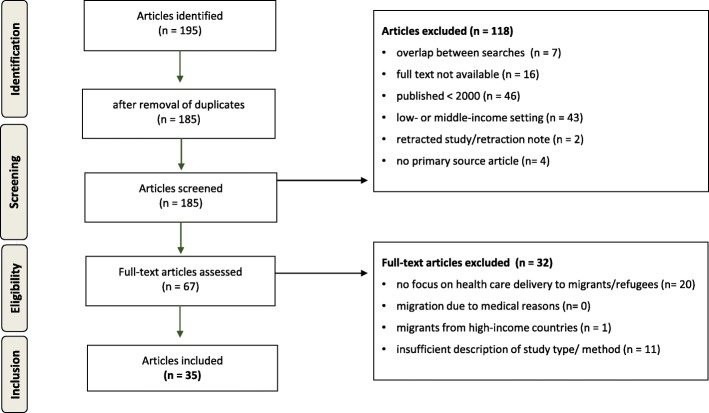
Flow diagram showing the process of study selection (adapted from [11]**)**

**Fig. 2:**
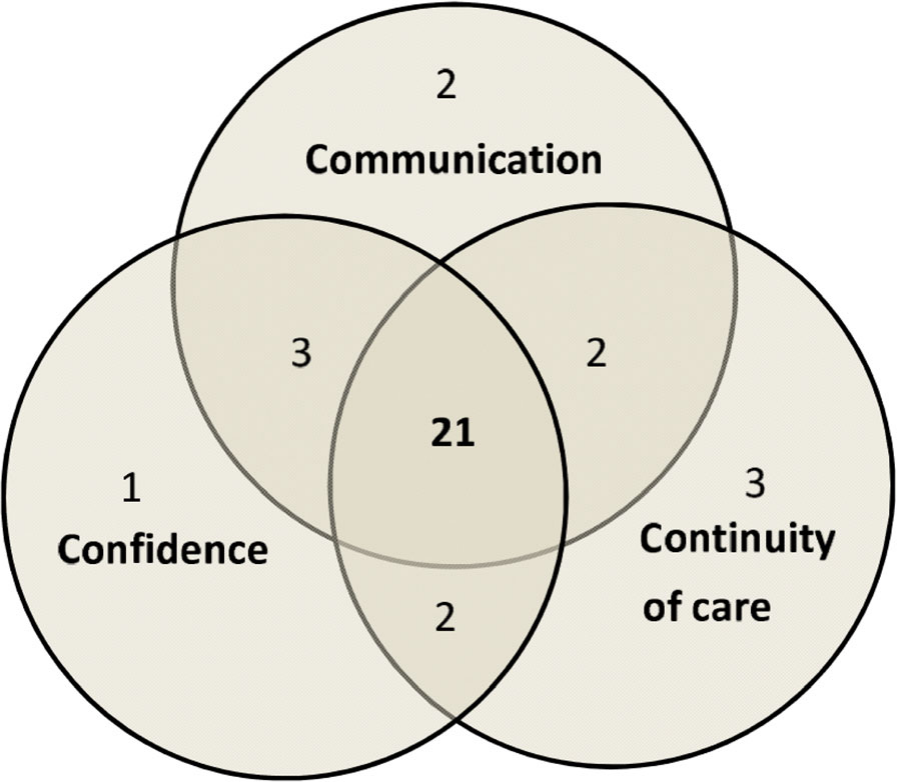
Venn-Diagram depicting the main categories discussed in the included studies. Of note only 34 studies are depicted as one study included did not mention any of the three main categories

**Fig. 3:**
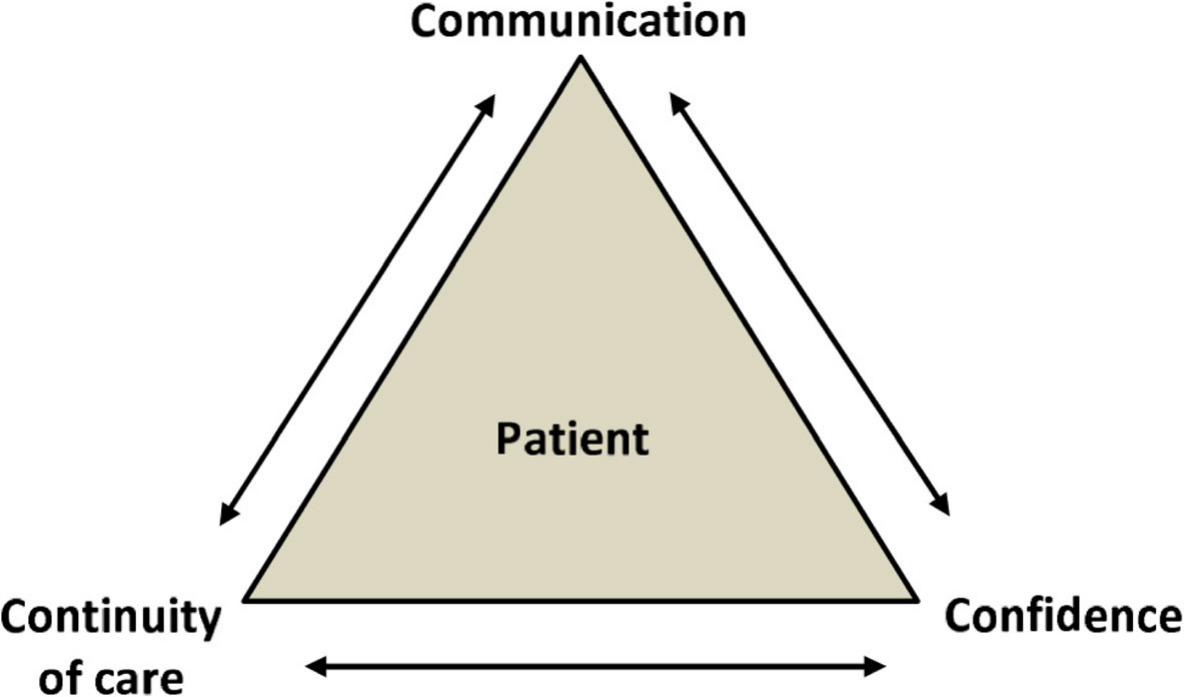
The 3C model summarizing the results of the analytical assessment. The three main categories influencing health care service delivery in refugees and migrants and are depicted, including their interrelation and the importance of a patient-centered perspective

A total of 195 search results of 185 publications were identified. Following screening and full text analysis 35 publications were included in the final assessment (Fig. 1). These comprised 12 cross-sectional studies, 17 qualitative studies, 3 good practice studies and 3 expert opinion articles. Most of the studies were from Australia, the US and the UK, followed by studies on Europe. Characteristics and the quality assessment of the included studies are summarized in Tables 1, 2 and 3. The three main topics discussed were: communication 29/35 (83%), continuity of care 28/35 (80%) and confidence 28/35 (80%). All but one study (34/35, 97%) included at least one of the three main topics and in 21/35 (60%) all three topics were mentioned (Fig. 2). The interrelation of the main categories was visualized in the 3 C Model (Fig. 3). Further topics mentioned were: context, collaboration, gender, holistic health care, social network and time.

**Table 1:** Characteristics of included studies. Descending order according to study type and study period

Study period	Country code	N participants	Details and focus of the study	C1	C2	C3	Other topics	First author & Reference
Cross-sectional studies								
2012–2015	US	363	Acceptance of mental health services in relation to age, gender, and country of origin	0	1	0	–	Ballard-Kang [13]
2013–2014	US	40	Refugees’ satisfaction with home health services visits	0	1	0	Home visits	Miner [14]
2008–2014	IT	151,311	Access to preventive health in national health surveys of Belgium, Italy, Malta, Portugal and Spain	0	0	0	Context	Rosano [15]
2001–2013	US	370	Analysis on initiation of antenatal care for migrant women who gave birth	1	1	1	Initiation visits	Kentoffio [16]
2008	US	810	Use of preventive medicine by Somali patients at US health facility	1	1	1	–	Morrison [17]
2005	UK	1611	Questionnaire survey of patients at an accident and emergency centre in London	1	1	0	–	Hargreaves [18]
2004	NL	580	Mental health service uptake of Turkish/Moroccan migrants and Dutch in NL	1	0	1	–	Fassaert [19]
2002	US	304	Impact of health counselling and health services on functional health outcomes of Sudanese youth arrived by Unaccompanied Refugee Minors Program	1	1	0	–	Geltman [20]
2001–2002	AU	199	East African children attending an immigrant health clinic for the first time	1	1	1	–	Cooke [21]
ns	US	70	Trauma related psychiatric symptoms in Bosnian refugees	0	0	1	–	Weine [22]
2011	CA	113	Language barriers in mental health; practitioners self-report survey in Montreal	1	1	1	–	Brisset [23]
2011	CA	41	Primary care practitioners performing modified Delphi consensus process on innovative strategies improving primary health care delivery to vulnerable populations	1	1	1	–	Pottie [24]
Qualitative studies								
2015–2016	CA	6	Interviews with health care providers about telemedicine for migrant health care delivery	1	1	1	Telemedicine	Sandre [25]
2012–2013	AU	64	Interviews/focus groups with Afghan parents who had recently had a child and health professionals on quality of care	1	1	1	Time, legal status	Yelland [26] [27]
2012–2013	AU	16	Evaluation of Australian mental health services from the perspective of young people with refugee background	1	1	1	–	Valibhoi [28]
2012	US	39	Focus groups with refugees from Iraq, Eritrea, Somalia, Bhutan on US health care	1	1	1	Context	Worabo [29]
2012	US & MX	33	Interviews on sexual health with indigenous women and nurses in migrant-sending and migrant-receiving communities	1	1	1	Gender	Espinoza [30]
2012	AU	87	Focus groups with mothers from refugee and migrant backgrounds on maternal and child health services	1	1	1	–	Riggs [31]
2010–2011	AU	115	Focus groups and key informant interviews with service providers experienced in young refugee’s mental health in Melbourne	1	1	1	–	Colucci [32]``
2010–2011	CA	15	Interviews with young mental health patients, caregivers and clinicians on quality of care	1	1	1	Collaboration	Nadeau [33]
2009–2010	UK	16	Interviews with NGO workers at HIV clinic on access to health services	0	1	0	–	Whyte [34]
2010	US & MX	15	Interviews with female patients and key informants in Mexico and California on access to and quality of sexual and reproductive health services	1	1	1	Context, gender	Deeb-Sossa [35]
2008–2009	UK	40	Interviews with adolescent refugees investigating school as convenient location for mental health services	0	1	1	Stigma, Collaboration	Fazel [36]
2005–2008	CA	47	Focus groups with Immigrants, refugees, non-status patients living with HIV/AIDS	1	1	1	Social events	Chen [37]
2005–2006	AU	34	Chinese mental health patients and providers on barriers to mental health care	1	0	1	–	Blignault [38]
2004–2006	AU	38	Focus groups and interviews with informants, providing health services to Afghans	1	0	1	–	Omeri [39]
ns	US	28	Interviews with Slavic emigres and key informants on chronic health conditions	1	1	1	Context	Van Son [40]
ns	UK	6	Interviews with Kurdish interpreters in UK	1	0	0	–	Green [41]
ns	US	20	Interviews with migrant farmworkers on health beliefs regarding their children	1	1	1	Context	Newton [42]
Good practice reports								
2017	AU	na	Characteristics to be collected in medical records to improve health care for migrants	1	0	0	Data collection	Yelland [43]
1987–2016	AU	na	Individualized mental health clinic	1	1	1	Holistic approach	Kaplan [44]
2010	AU	na	Narrative medicine in refugee mental health	1	1	1	–	Benson [45]
Expert opinions								
2016	CA	na	Description of migrant health care delivery in Canada	1	1	1	Context	Rahman [46]
2009	IT	na	Expert analysis of reason for results of Swedish cohort study on psychotropic substance use	0	1	1	–	Nose [47]
2002	AU	na	Problems refugees face in accessing effective health care; ways in which health services can respond	1	1	1	–	Lamb [48]
TOTAL				**29**	**28**	**28**		

AIDS: acquired immune deficiency syndrome; C1: Communication; C2: Continuity of Care; C3: Confidence; HIV: Human immunodeficiency virus; na; not applicable; N: number

*NGO*: Non-governmental organisation

**Table 2:** Quality assessment tool for cross sectional studies: Newcastle-Ottawa Scale (adapted from Modesti PA et al. [21])

First author & reference	Selection				Comparability	Outcome		Score
	Representativeness of the sample	Sample size justified	Non-respondents	Ascertainment of exposure (max**)	Confounding controlled (max**)	Outcome assessment (max**)	Statistics	Total
Ballard-Kang [13]	*	*	*	–	*	**	*	7/10
Miner [14]	*	*	na	**	na	**	*	7/10
Rosano [15]	*	*	–	**	**	**	*	9/10
Kentoffio [16]	*	*	na	**	**	**	*	9/10
Morrison [17]	*	*	na	**	**	**	*	9/10
Hargreaves [18]	*	*	*	*	**	*	*	8/10
Fassaert [19]	*	*	*	**	**	*	*	9/10
Geltman [20]	*	*	*	**	**	*	*	9/10
Cooke [21]	*	–	–	**	na	*	–	4/10
Weine [22]	–	*	–	**	**	*	*	7/10
Brisset [23]	*	*	–	**	–	*	*	6/10
Pottie [24]	*	*	*	**	na	*	*	7/10

**Table 3:** Quality assessment tool for qualitative studies according to CASP [11]

First author & reference	Clear aim	Method appropriate	Study design	Recruitment	Data collection	Relationship researcher- participants	Ethics	Rigorous analysis	Findings	Valuable results	Score
Sandre [25]	*	*	–	*	*	–	*	*	*	*	8/10
Yelland [26] [27]	*	*	*	*	*	–	*	*	*	*	9/10
Valibhoi [28]	*	*	*	*	*	–	*	–	*	*	8/10
Worabo [29]	*	*	*	–	*	–	*	*	*	*	8/10
Espinoza [30]	*	*	*	*	*	–	*	–	*	*	8/10
Riggs [31]	*	*	*	*	*	–	*	*	*	*	9/10
Colucci [32]	*	*	*	*	*	–	*	–	*	*	8/10
Nadeau [33]	*	*	*	*	*	–	*	*	*	*	9/10
Whyte [34]	*	*	–	–	–	–	–	–	*	*	4/10
Deeb-Sossa [35]	*	*	*	*	*	–	*	*	*	*	9/10
Fazel [36]	*	*	*	*	*	–	*	*	*	*	9/10
Chen [37]	*	*	*	*	*	–	*	*	*	*	9/10
Blignault [38]	*	*	*	*	*	–	*	*	*	*	9/10
Omeri [39]	*	*	*	*	*	*	*	*	*	*	10/10
Van Son [40]	*	*	*	*	*	*	*	*	*	*	10/10
Green [41]	*	*	*	*	–	–	*	*	*	*	8/10
Newton [42]	*	*	*	*	*	*	*	–	*	*	9/10

### Communication

Communication was the topic most commonly mentioned. Generally, communication is required for the understanding of the reason for the presentation of a patient and allows to exchange information about symptoms, presumed diagnosis, diagnostic tests required, treatment and Prognosis [46]. Speaking the same language was mentioned as a gateway to health care [39].

Communication was reported to be challenging for health care providers and patients alike [25]. Health care workers stated that a different language was an obvious barrier and may lead to misunderstanding or lack of understanding [19, 25].

In a study including Mexican women in the United States, Espinoza et al. described that communication barriers resulted in reduced utilization of health services [30]. In host countries not offering free language courses to migrants and refugees, patients found it difficult to improve their communication skills in the local language [39]. If free language courses were available time was required to learn a language to be able to explain health problems appropriately as shown in a study including Sudanese boys in the US [20]. Beyond basic communication skills were required to express feelings and beliefs and particularly challenged patients with mental health disorders [20].

Interpreter services therefore played a key role in medical consultations [19, 45]. The availability of interpreters was described as directly increasing adequate health service provision and use among migrants and refugees. This resulted in a decrease of social vulnerability [38, 40]. Different types of interpreter services including phone or video interpreters, in-person professional or non-professional interpreters were mentioned. Quality regarding the accuracy of the translation was considered important by health care providers and patients. In a qualitative study from Montreal, health care providers preferred professional to non-professional interpreters. They were considered to offer the advantages of knowledge of the health care system, specialized health vocabulary and enabled them to fulfil their professional responsibilities [23]. The absence of system flexibility to allocate time for interpreter-mediated appointments was described as problematic [27]. The consistent availability of a preferred professional interpreter enhanced provider-patient communication and relationship [23, 44]. A briefing and debriefing before and after the consultation with the health care provider was considered helpful to prevent misunderstandings and help professional interpreters to cope with challenging topics [41]. As opposed to health care providers who were reluctant to use the patients´ family members or friends as interpreters, migrants and refugees generally trusted them and appreciated their help [13, 23]. Beyond transmission of information, the interpreting individual was reported to influence relationships, judgments and decisions [28].

According to Yelland et al. systematic assessment and documentation of the need for an interpreter should be included in the provision of migrant health care [43]. A mismatch of patient and interpreter for gender, age, dialect or ethno-cultural factors was considered challenging, as specifically pointed out by a study on the access to mental health facilities by young people with a refugee-background in Australia [32]. Sometimes the use of an interpreter was not only considered positive, as the need for translation frustrated older migrants and refugees who had been functioning independently in their home countries .

### Continuity of care

Continuity of care was the second most common topic mentioned in the studies. Most important factors influencing the continuity of care were: a) information and education for migrants and refugees about the health care system of the host country b) ease of access of health facilities, c) integration of medical appointments into the personal schedule of migrants and refugees and d) collaboration of different institutions, ensuring minimal loss of health care information.

Knowledge on the health care system of the host country was considered integral to become health literate and to make the continuity of care possible. In case such information was not provided, patients were more likely to present to an emergency department [18, 29]. Several concepts such as general practitioners and preventive health may be new to migrants and refugees and required careful explanation [29]. Unfamiliar with the concept of prevention visits and systematic screening of diseases, patients saw no reason to attend an appointment “when there [was] no health problem” [29].

An important factor limiting the continuity of care was access to health care services. For example being located in remote facilities may complicate the journey to medical appointments [25]. Generally, refugees and migrants may have no access to private transportation soon after arrival and depend on public transport use, which they found challenging [31]. After arrival, the process of adjusting to a new culture is complex and stressful and may stand in the way of attending health care appointments [49]. Integration of health care visits into other appointments related to the asylum process or education enhanced the continuity of care. For example, in a study from the UK young adults preferred having their mental health service at school so they could easily integrate treatment into their class schedule [36].

Coordination between different health care providers was reported as a further way to enhance attendance to medical appointments and reduce health information loss [32, 33, 37]. A clear guidance on why, where and when to continue treatment was essential for the continuity of care [37]. Community members, knowledgeable in the health care system of the host country were described as important resources to bridge between refugees and migrants and public services [32]. Ensuring regular attendance of follow-up appointments may require holistic strategies including home visits [14, 31], arranging transportation or using reminder phone calls for appointments [44]. Models allowing flexibility in the scheduling of consultations were particularly welcomed by adolescent patients living in Canada, allowing them to adapt the timing of their medical appointments spontaneously [33].

### Confidence

Confidence was the third most common topic mentioned. It consisted of two main parts: the development of trust in someone or something and the ability to control a situation [50–52]. Health care providers and patients agreed that finding a common ground was required to establish a trustful relationship and this was a bilateral process requiring mutual education [45]. In a study including migrant farm workers in the US, a respectful health care provider was described as a person greeting by name, listening, engaging in conversation, and allowing family members to stay with the patient during procedures [42]. Respect shown by health care providers was essential for migrants and refugees with traumatic experiences [28, 45]. If the patient had been tortured by health care providers or if they were part of the persecuting regime, a lack of trust in institutions and professionals, including hospitals and people in any kind of uniform resulted [28, 45, 48]. One study described that respectful treatment was the most important criteria for migrants and refugees when choosing a health care provider for their children [42]. In case no trustful relationship was established, patients used traditional medicine and trusted “their own resources” from their community for health related questions [40].

For migrants and refugees health literacy education and training about the country’s health care system was key [45]. Information about the new health care also lead to familiarity and ultimately trust in the system. Studies pointed out that trust in the new system and concepts was needed to integrate them into own concepts. The inclusion of family members and friends in health care decisions was further increasing a trustful relationship and confidence [13, 22].

For health care providers education on the background of refugees and migrants and development of intercultural skills has been described as enhancing confidence in patients [22]. Intercultural trainings help to acknowledge the culturally different roles of religion and spirituality and to understand the potentially different importance of family structure, relations and gender roles [46].

A trustful relationship was also required before patients disclosed delicate health needs. In one study, a nurse reported that it took her one year to develop a trusting relationship before the mother disclosed domestic violence [31]. In another study a participant stated that the real treatment was not medical but friendship: “The healing power of love, humor and kindness should not be ignored. It is rare for these elements to be added to our evidence base, but for people who have suffered dreadfully at the hands of our fellow human beings these may be rare commodities” [45]. Previous experiences influenced perception of health service delivery in the host country. Positively associated similarities between health care delivery in home and host countries improved access to care and satisfaction [29, 30].

Besides the development of trust into someone or something, the feeling of being in control was important for confidence. To be able to control health care decisions, understanding facts and applying own health beliefs and priorities for decision making is required. The ability to feel self-sufficient and being ahead of decisions was described as particularly important in migrant mental health [45]. Language barriers or interpreter requirement impeded the feeling of control over health care decisions [40]. In a study on maternal and child health conducted in Melbourne, a Sudanese mother needed three days to make an appointment by phone due to a lack of confidence in her ability to manage health care visits by phone, despite basic knowledge of the local language [31]. In some cultures, the ability to have control over health care is gender-dependent. In two studies on Mexican migrants living in the United States, male family members decided on women’s sexual and reproductive health. These gender structures affected women’s ability to choose and therefore control their sexual health, despite official laws of the host country stating otherwise [30, 35]. Confidentiality by the health care providers and interpreters regarding personal information was also considered important for confidence [30, 32]. Simple behavioral patterns of health workers may help refugees and migrants to gain control and feel confident. For migrant farmworkers in the US, it was considered helpful if health care providers avoided frequent questions about the legal status [42].

Other studies emphasized collaborative decision making [32, 33]. On a structural level, confidence was improved if migrants and refugees were empowered to take control for example in planning and deciding about the right location of health services for their communities [39].

### Context challenges

Further to the three main topics, all studies mentioned that health care delivery was embedded in the regional context. Consequently communication, continuity of care and confidence were influenced by the specific context of the host countries. Main factors determining the context were legal, financial, geographical and cultural aspects specific to the host countries, as illustrated in the extension of the 3C model (

**Fig. 4:**
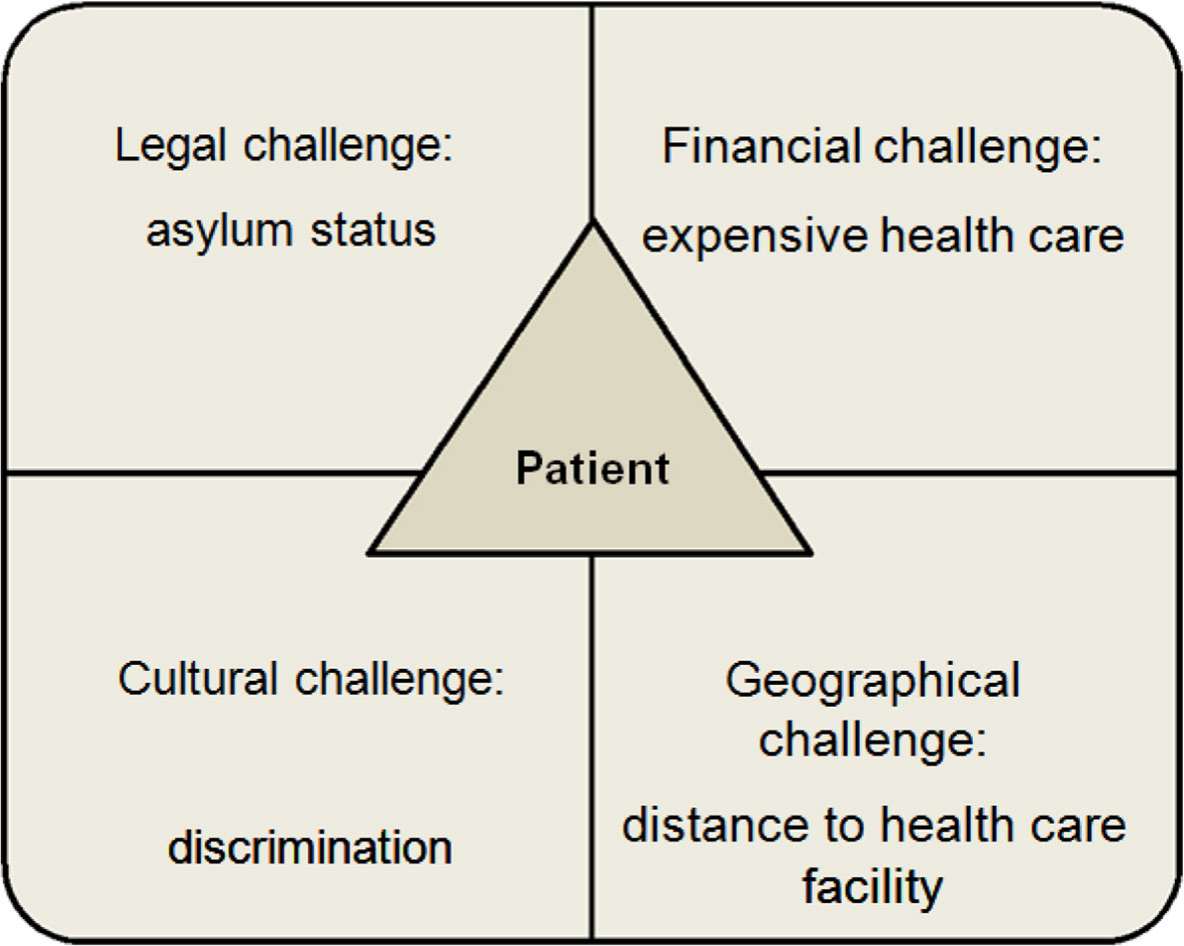
Extension of the 3C model, summarizing the main context challenges

Figure 4) [53].

First, legal aspects have a considerable influence in many settings as shown by migrants and refugees views’ that citizenship is a prerequisite to access health care [34, 37, 40]. Reciprocally, concerns about legal status affected their health and wellbeing [28, 36, 39]. Second, cost and limited finances deterred migrants and refugees from utilizing health care services [37]. However in one study investigating the effect of near-universal health coverage for refugees and migrants, they still showed an increased risk for delayed prenatal care [16]. This underlines the importance of other factors, like concerns about housing or family separation that kept refugees and migrants from seeking health care [32, 47]. Third, geographical aspects are also important as migrants and refugees living in rural areas were challenged to access health facilities far away from their housing [48].

Finally, the cultural aspect including intolerance of the host communities towards migrants in general or religious beliefs posed important barriers, as shown in a study including Afghan refugees in Australia [39].

## Discussion

Communication, continuity of care and confidence were identified as the three main factors influencing migrant and refugee health care delivery in high income countries. The three categories are closely interrelated, and reciprocal influence was frequently described (Fig. 3). Communication has been recognized as the key starting point allowing to build-up confidence between the health care provider and the patient. This further enhanced continuity of care. Importantly, studies pointed to the fact that the use of interpreters may be viewed differently by health care workers and patients. Health care workers preferred standardized settings including professional interpreters as these are perceived to convey information in the best way, whereas refugee and migrant patients however felt that family members and friends are convenient interpreters as they are trusted persons. These examples show the complexity of and delicate balance in the challenges in migrant and refugee health care delivery within the 3C model. Flexibility is therefore required for systems and health care workers to permit more than standard solutions in order to allow patient-centered care.

### Comparision of views of health workers and patients

It is important that health care workers are aware of discrepancies in their perceptions and those of the refugee and migrant patients. Studies have shown that the health workers´ critical self-reflection is a prerequisite for good quality care and particularly important when providers and patients are from different sociocultural backgrounds [54]. Critical self-reflection enables health care workers to discover dissimilar concepts, for example in the migrants´ and refugees´ views of preventive care. Interestingly, our systematic review showed that in many instances the views of health care workers and refugee and migrant patients were however similar in many key aspects including the importance of confidence and mutual learning.

The specific context of the host country largely determines the conditions, in which communication, continuity of care and confidence can be provided. A recently published qualitative literature review focuses on the views of primary health care professionals on health care delivery to asylum-seekers and refugees. The results highlight the importance of the health system level and the level of asylum and resettlement for the provision of care [55]. Legal factors defined by international, regional and national policies have a major impact on access to health care. For example, non-EU migrants experienced the largest gap in access to preventative health care compared to the resident population or the EU migrants [15]. As contributing factors, the lack of targeted health policies focusing on the most vulnerable was identified. Furthermore, in countries where the legal status directly defines the extent by which health services are accessible, negative health implications for refugees and migrants are the consequence [56]. Absence of continuous financial coverage for health expenditures on refugees and migrants also directly limited the continuity of care provided [57]. Cooperation and coordination between different stakeholders was considered important by a systematic review assessing different health care models for refugees [58]. A recent study described the lack of standardization in health assessments, data collection and health information regarding potential infectious diseases of migrants and refugees in Europe. This lowered the health system performance and the preparedness for epidemics [59].

A limitation with this study is that it deals with a heterogeneous group of migrants and refugees, similar to the one described in detail by the UCL-Lancet Commission on Migration and Health [60]. We were able to exclude studies on “skilled migrants” as this is a well-defined group with clearly different predispositions and different access to health care. We were unable to analyze results separately for different specific situations or groups of migrants, potentially neglecting significant discrepancies between groups and local conditions. However, this heterogeneity mirrors the reality in which health care delivery to migrants and refugees takes place and increases the likelihood that the 3C model is relevant for the diversity of patient’s backgrounds and settings. Another limitation is that the studies included only refugees and migrants who attended official health care services. Those not presenting to any health care services or to unofficial services are not represented. In addition, this review included only studies published until December 2017. Therefore, the data and the model will need to be reevaluated in the light of evidence published after this date. We also acknowledge that delivery of health care to refugees and migrants is complex, involving a myriad of different aspects and stakeholders. The reduction to a model including only three categories may result in an oversimplification. It is important to recognize that each category itself contains a large range of aspects, for which we are unable to include an in-depth discussion of each. In addition, some aspects may be discussed under one category but equally influence others. The condensation of the current evidence to the three categories was done with methodological rigor and based on current best research methodology including the mixed methods research synthesis framework.

## Conclusion

The 3C model summarizes the major challenges in migrant health care delivery, namely communication, continuity of care and confidence. We also found how important the context is and identified four key factors namely legal, financial, geographical and cultural challenges. The model gives a comprehensive, patient-centered summary of important areas relevant to support clinicians in their day-to-day practice, in training of health care providers and to guide priority setting in migrant health policies. Coordination and cooperation of stakeholders, relevant to migrant health care delivery is needed to ensure efficient action and to maximize the impact of available human and financial resources.

## Additional file

Additional file 1: **Figure S1.** Mixed methods research synthesis used for this review (adapted from Heyvaert M et al. ^9^) (DOCX 20 kb)

## Data Availability

The datasets used and/or analyzed during the current study are available from the corresponding author on reasonable request.

## Abbreviations

AIDS: Acquired immune deficiency syndrome
AU: Australia
CA: Canada
HIV: Human Immunodeficiency Virus
IT: Italy
MMRS: Mixed methods research synthesis framework
MX: Mexico
NGO: Non-governmental Organisation
NL: Netherlands
PRISMA: Preferred reporting items for systematic reviews and meta-analyses
UK: United Kingdom
US: United States of America
WHO: World Health Organisation
