# Supplementary material for: A systematic literature review of reported challenges in health care delivery to migrants and refugees in high-income countries - the 3C model
Source: BMC Public Health. 2019 Jun 14;19:755. doi: 10.1186/s12889-019-7049-x (PMC6567460; doi:10.1186/s12889-019-7049-x)

Additional file 1: **Figure S1**. Mixed methods research synthesis used for this review (adapted from Heyvaert M et al ^9^)


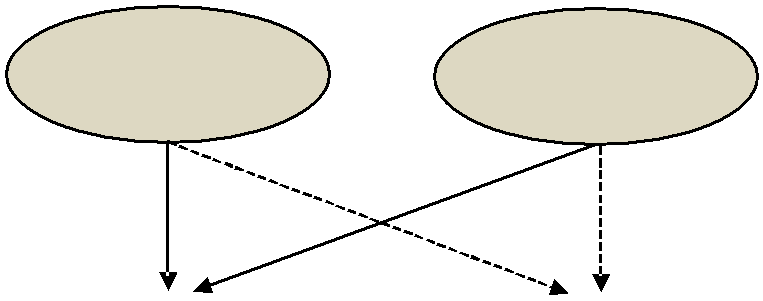


| Primary level studies | Qualitative | Quantitative |  |
| --- | --- | --- | --- |
|  | studies | studies |  |
|  |  |  |  |

| Synthesis studies | Qualitative synthesis |  | Quantitative synthesis |
| --- | --- | --- | --- |
|  |  |  |  |
|  | : done in this study | : not done in this study | |


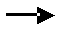

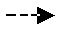

Supplement: Supplementary file 1 — Figure S1. Mixed methods research synthesis used for this review (adapted from Heyvaert M et al. 9) (DOCX 20 kb) [file 12889_2019_7049_MOESM1_ESM.docx]
